# GlyCEST: Magnetic Resonance Imaging of Glycine—Distribution in the Normal Murine Brain and Alterations in 5xFAD Mice

**DOI:** 10.1155/2021/8988762

**Published:** 2021-12-30

**Authors:** Ken Ohno, Masaki Ohkubo, Bingwen Zheng, Masaki Watanabe, Tsuyoshi Matsuda, Ingrid L. Kwee, Hironaka Igarashi

**Affiliations:** ^1^Center for Integrated Human Brain Science, Brain Research Institute, University of Niigata, Niigata 951-8585, Japan; ^2^Department of Radiological Technology, Faculty of Medical Technology, Niigata University of Health and Welfare, Niigata 950-3198, Japan; ^3^Department of Radiological Technology, School of Health Sciences, Faculty of Medicine, University of Niigata, Niigata 951-8518, Japan; ^4^Time Medical Ltd., Hong Kong Science & Technology Park, Hong Kong, China; ^5^Division of Ultrahigh Field MRI, Institute for Biomedical Sciences, Iwate Medical University, Iwate 028-3694, Japan; ^6^Department of Neurology, University of California Davis, CA 94553, USA

## Abstract

The glycine level in the brain is known to be altered in neuropsychiatric disorders, such as schizophrenia and Alzheimer's disease (AD). Several studies have reported the *in vivo* measurement of glycine concentrations in the brain using proton magnetic resonance spectroscopy (^1^H-MRS), but ^1^H-MRS is not capable of imaging the distribution of glycine concentration with high spatial resolution. Chemical exchange saturation transfer magnetic resonance imaging (CEST-MRI) is a new technology that can detect specific molecules, including amino acids, in tissues. To validate the measurements of glycine concentrations in living tissues using CEST from glycine to water (GlyCEST), we extracted the brain tissues from mice and performed biochemical tests. In wild-type C57BL/6 mice, GlyCEST effects were found to be higher in the thalamus than in the cerebral cortex (*P* < 0.0001, paired *t*-test), and this result was in good agreement with the biochemical results. In 5xFAD mice, an animal model of AD, GlyCEST measurements demonstrated that glycine concentrations in the cerebral cortex (*P* < 0.05, unpaired *t*-test) and thalamus (*P* < 0.0001, unpaired *t*-test), but not in the hippocampus, were decreased compared to those in wild-type mice. These findings suggest that we have successfully applied the CEST-MRI technique to map the distribution of glycine concentrations in the murine brain. The present method also captured the changes in cerebral glycine concentrations in mice with AD. Imaging the distribution of glycine concentrations in the brain can be useful in investigating and elucidating the pathological mechanisms of neuropsychiatric disorders.

## 1. Introduction

Glycine, which is the simplest amino acid found in the proteins of living organisms, is known to act as an inhibitory neurotransmitter in the brainstem and spinal cord of the central nervous system [1, 2]. In contrast, it acts in the cerebrum as a coagonist for the N-methyl-D-aspartate (NMDA) receptor, an ionotropic glutamate receptor [3, 4]. The NMDA receptor controls the influx of calcium into neurons and is essential for synaptic transmission, neuroplasticity, and learning and memory mechanisms [5, 6]. Activation of NMDA receptors requires the binding of glutamate and glycine to their binding sites in their respective subunits [7]. The extracellular glycine concentration is altered by brain activity and is mainly regulated by astrocytic glycine transporters [8]. Glycine concentration is thought to be related to NMDA receptor function, and glycine is sometimes used for the treatment of schizophrenia [9]. Considering the essential roles of glycine in brain metabolism and function, better knowledge about glycine distribution *in vivo* may further elucidate its functions under physiological and pathological conditions.

The glycine concentration in the brain is known to be altered in neuropsychiatric disorders such as schizophrenia and Alzheimer's disease (AD) [10–12]. In patients with AD, blood and brain glycine concentrations differ from those in healthy subjects [10, 11], although other studies have shown that glycine concentrations are unchanged in patients with AD [12]. We hypothesized that these inconsistent data could be caused by variations in glycine concentrations in the different areas of the brain. Thus, the measurement of regional glycine concentrations and imaging of glycine concentration distributions in the brain may provide knowledge on the pathogenesis of AD and facilitate the staging of this disease.

To measure the concentration of glycine in living tissues, it is necessary to extract the tissues and measure the amount of protein using high-performance liquid chromatography (HPLC) or mass spectroscopy. Several studies have reported the *in vivo* measurement of glycine concentrations in the brain using proton magnetic resonance spectroscopy (^1^H-MRS) [13–15], but this technique is not capable of showing the glycine concentration distribution with high spatial resolution. Chemical exchange saturation transfer magnetic resonance imaging (CEST-MRI), which measures the exchange of target metabolite protons to water molecules and calculates the concentration of metabolites [16], can be used to obtain the glycine concentration distribution with high spatial resolution.

The purpose of this study was to establish a method to measure the distribution of glycine using the CEST-MRI technique (GlyCEST) and evaluate the application of this method in diseases. We first validated GlyCEST using a phantom containing solutions of known glycine concentrations. Subsequently, we applied the method to the murine brain *in vivo*. Finally, the 5xFAD mouse model of AD [17] was used to verify the capability of this method to detect changes in glycine concentrations in the brains of mice with AD.

## 2. Materials and Methods

### 2.1. Phantom Preparation

To study the linearity of concentration-dependent GlyCEST effects, we used solutions with varying glycine concentrations (0, 2.5, 5.0, 7.5, and 10.0 mM) in phosphate-buffered saline, adjusted to pH 7.0 using 1 M HCl or 1 M NaOH. These solutions were added to 10.8 mm inner diameter tubes that were immersed in a 33.7 mm inner diameter glass tube filled with phosphate-buffered saline at pH 7.0.

### 2.2. Experimental Animals and Setup

This study was approved by the Institutional Animal Care and Use Committee of Niigata University (SA00849) and conducted in accordance with the Guidelines of the US National Institutes of Health regarding the care and use of animals for experimental procedures [18]. We utilized seven homozygous 5xFAD transgenic male mice expressing five mutant human genes associated with AD (APPSwFlLon, PSEN1^*∗*^M146L^*∗*^L286V) and 6799Vas/Mmjax [17] at 16-17 months of age, as well as 16 C57BL/6 wild-type (WT) male mice at 2-3 months of age. Breeding progenitors were purchased from Jackson Laboratory (Bar Harbor, ME, USA). All animals were maintained under standard laboratory conditions with a 12 h/12 h light/dark cycle with access to food and water ad libitum. The 5xFAD mice were genotyped by polymerase chain reaction analysis of DNA obtained from tail biopsies.

In a first set of experiments, we assessed whether GlyCEST could detect regional differences in glycine concentrations. Nine C57BL/6 WT mice were used for these GlyCEST measurements. Mice were anesthetized using isoflurane (induction with 3%–4%, followed by 1.2%–1.5% maintenance) mixed with 30 : 70 O_2_ : N_2_O at 2 L/min and placed into the MRI scanner using head fixation with ear and tooth bars. The rectal temperature of the animal was maintained at 37 ± 0.5°C using a custom-made temperature-controlled air conditioning system. After MRI measurements, brains were harvested to correlate the MRI data with biochemical measurements. The extracted brains were cut into 4 mm thick coronal slices (±2 mm from the center of the MR imaging slab) and divided into the cortex and the thalamus.

In a second set examining the reproducibility of GlyCEST data, we measured GlyCEST twice on the same day in the same mice. Five C57BL/6 WT mice were used for these reproducibility measurements.

In a third set, we assessed GlyCEST effects in 5xFAD mice (*n* = 7) and C57BL/6 WT mice (*n* = 7). The GlyCEST measurements and brain sample preparation protocols were identical to those used in the first set of experiments.

### 2.3. HPLC Glycine Measurements

The specimens extracted from murine brains were measured using HPLC. The collected tissue samples were dissolved in 5 mL of 80% methanol. The samples were centrifuged (3,000 rpm for 15 min), and 5 *μ*L of the supernatant was analyzed by HPLC. The measurement was performed using an Agilent 1100 series HPLC-FLD system (Agilent Technologies, Wilmington, USA) with a fluorescence detector and an Agilent Poroshell 120 EC-C18 (3.0 × 150 mm, 2.7 *μ*m) column. To 5 *μ*L of each sample, 5 *μ*L of boric acid solution, 0.5 *μ*L of ortho-phthalaldehyde/3-mercaptopropionic acid solution, and 0.5 *μ*L of 9-fluorenylmethyl chloroformate solution were added and mixed. For sample injection, Na_2_HPO_4_ (5 mM, pH 7.6) was used as the mobile phase, and the pump flow rate was set to 0.5 mL/min with a column temperature of 40°C [19]. The Agilent “ChemStation” software (ChemStation for LC 3D systems, Rev B.04.02 SP1) was used to control the system and analyze the data.

### 2.4. MRI Measurements

MRI was performed using a 16 cm bore 7-T horizontal magnet (Magnex Scientific, Abingdon, UK) with an Agilent Unity-INOVA-300 system (Agilent Inc., Palo Alto, CA, USA) equipped with an actively shielded gradient. A custom-made volume transmitter and a quadrature surface receiver proton coil set (Takashima Seisaku-syo, Hino, Japan) were used for the MRI measurements. The imaging protocol was carried out in the following order: (1) localizers (three planes), (2) T2-weighted spin-echo morphological images (T2WI), and (3) GlyCEST. The total image acquisition time for each mouse was 43 min. GlyCEST imaging consisted of a 418 ms saturation pulse train (consisting of ten 40 ms Gaussian-windowed saturation pulses with a 2 ms interpulse delay) at B1rms = 5 *μ*T, followed by centric ordered snapshot fast low-angle shot (FLASH) acquisition. Other imaging parameters were as follows: slice thickness = 2 mm, flip angle = 20°, matrix = 128 × 64, repetition time (TR) (for FLASH) = 4.37 ms, echo time (TE) = 2.20 ms, total TR = 5,000 ms, and signal averages = 4. Raw CEST images were acquired at varying offset frequencies of saturation pulses, from −5 ppm to 5 ppm (−1,500 Hz to 1,500 Hz on 7-T MRI) with a step size of 60 Hz. To map B0 inhomogeneity, water saturation shift referencing images with 100 ms Hanning windowed saturation pulses at B1rms = 0.5 *μ*T were acquired at saturation offset frequencies ranging from −0.5 ppm to 0.5 ppm for the water peak, with a step size of 6 Hz.

### 2.5. GlyCEST (%) Computations

In GlyCEST imaging, a saturation pulse that selectively suppresses a specific frequency is applied. The resonance frequency of glycine differs from the water peak by +2.85 ppm. When the irradiation position was +2.85 ppm, glycine was saturated, and the signal intensity of the MR image was reduced due to the proton exchange between water and glycine molecules. From the signal intensities of *M*_+2.85 ppm_ and *M*_−2.85 ppm_ at + 2.85 ppm and −2.85 ppm, GlyCEST (%) = (*M*_−2.85 ppm_ − *M*_+2.85 ppm_)/*M*_−2.85 ppm_ × 100 was calculated as an index to quantitatively evaluate the CEST effect. The calculation of GlyCEST (%) on a pixel-by-pixel basis created the glycine concentration map (GlyCEST map). All data processing and analyses were performed using MATLAB (Version 2020a, MathWorks, Natick, MA, USA). Region of interest (ROI) analysis of the GlyCEST map utilized ImageJ software (Version 1.53a, National Institutes of Health, https://imagej.nih.gov/ij/index.html). Five circular ROIs, corresponding to tubes containing 0, 2.5, 5.0, 7.5, and 10.0 mM glycine, were used for the glycine phantom. Four anatomical ROIs, corresponding to the whole cortex, parietal cortex, temporal cortex, and thalamus, were manually drawn on the images of the mouse brain.

### 2.6. Statistical Analyses

Statistical analysis was performed using GraphPad Prism version 9 (GraphPad Software, La Jolla, CA, USA). Pearson's product-rate correlation coefficient was used to analyze the correlation between glycine concentrations and GlyCEST (%) and between HPLC and GlyCEST data. The paired *t*-test was used to compare the means of glycine concentrations and GlyCEST (%) values in the cortex and thalamus in C57BL/6 WT mice. To evaluate the reproducibility of GlyCEST measurements, we calculated the intraclass correlation coefficient (ICC) [20] between the first and second MRI measurements of GlyCEST (%) values in the cerebral cortex and thalamus of the same C57BL/6 mice. ICC quality was judged as follows: ICC < 0.4 poor, 0.40–0.60 fair, 0.60–0.75 good, and 0.75–1 excellent [21]. The unpaired *t*-test was used to compare the means of regional GlyCEST measurements in WT and 5xFAD mice. A *P* < 0.05 was considered statistically significant. All data are shown as mean ± standard deviation.

## 3. Results

### 3.1. Correlation between GlyCEST (%) and Glycine Concentrations

**Figure 1:**
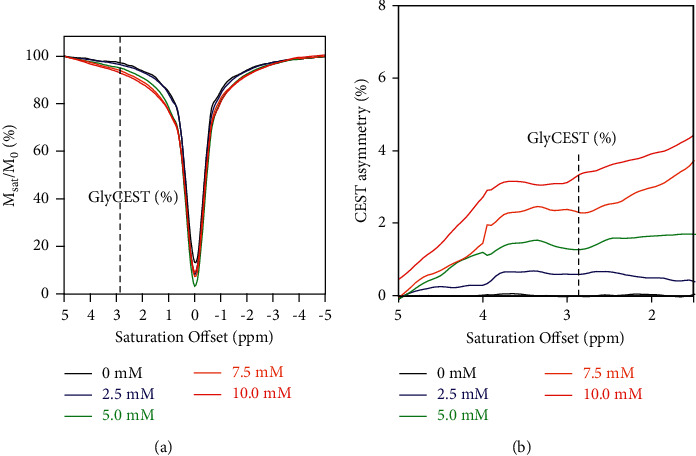
GlyCEST effects. (a) z-spectra of a phantom containing different glycine concentrations (pH 7.0). (b) CEST asymmetry at various saturation offsets for different glycine concentrations. The same computation was used to determine GlyCEST (%).

**Figure 2:**
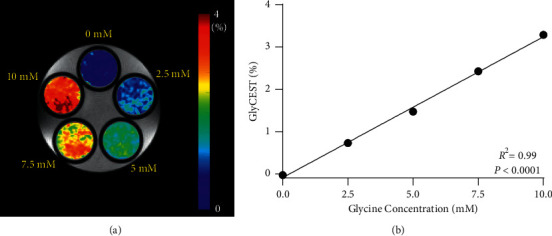
(a) GlyCEST map of a phantom. (b) Significant linear correlation between GlyCEST (%) and glycine concentration.

The z-spectra, giving the ratios of water signal intensities with (*M*_sat_) and without (*M*_0_) selective saturation, showed that the *M*_sat_/*M*_0_ ratio decreased with glycine concentrations increasing from 0 to 10.0 mM (Figure 1(a)). The z-spectra showed an asymmetry between the positive and negative saturation offsets from the water resonance; the CEST asymmetry in each saturation offset was evaluated by the same computation as used for obtaining GlyCEST (%) (Figure 1(b)). The CEST asymmetry increased with increasing glycine concentrations. For each pixel, GlyCEST (%) at the saturation frequency of 2.85 ppm was calculated, and a GlyCEST map was created (Figure 2(a)). GlyCEST (%) increased linearly with increasing glycine concentrations and was significantly correlated with the glycine concentration (*R*^2^ = 0.99 and *P* < 0.0001) (Figure 2(b)).

### 3.2. Correlation between GlyCEST (%) and Glycine Concentrations Determined by HPLC

**Figure 3:**
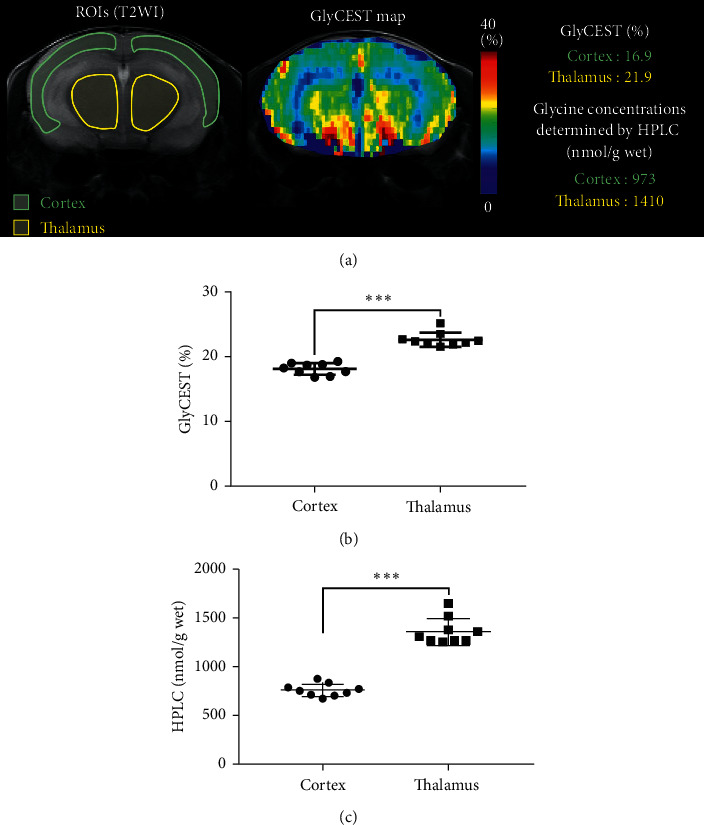
GlyCEST effects and glycine concentrations in C57BL/6 wild-type (WT) mice (*n* = 9). (a) T2-weighted spin-echo image (T2WI) showing regions of interest (ROIs) in a coronal brain slice (left panel) and the corresponding GlyCEST map (center panel). The corresponding GlyCEST (%) and glycine concentrations detected by high-performance liquid chromatography (HPLC) analysis in the cortex and thalamus are also shown (right panel). (b) GlyCEST (%) in the cortex and thalamus measured in T2WI-defined ROIs. (c) Glycine concentrations in the cortex and thalamus detected by HPLC. In both GlyCEST and HPLC measurements, the glycine concentration in the thalamus is higher than that in the cortex. Data were analyzed using the paired *t*-test (^∗∗∗^*P* < 0.0001).

WT mice (*n* = 9) were imaged for GlyCEST, and ROIs were placed in the cortex and thalamus based on the corresponding regions on T2WI images (Figure 3(a)). Following GlyCEST imaging, the cortex and thalamus of the murine brains were extracted for HPLC analyses. The GlyCEST (%) values were significantly higher in the thalamus than in the cortex (*P* < 0.0001) (Figure 3(b)), which is consistent with the glycine concentrations measured by HPLC (Figure 3(c)). This finding indicates that GlyCEST can detect local differences in cerebral glycine concentrations.

Some WT mice (*n* = 5) were imaged twice for GlyCEST (Supplementary Figure S-1(a)), and ROIs were placed in the cerebral cortex and thalamus (Supplementary Figure S-1(b)). Both cortical and thalamic GlyCEST (%) values showed excellent ICCs (0.88 in both the cortex and the thalamus) (Supplementary Table S-1). These results indicate that GlyCEST measurements are reliable.

### 3.3. Regional GlyCEST Effects

**Figure 4:**
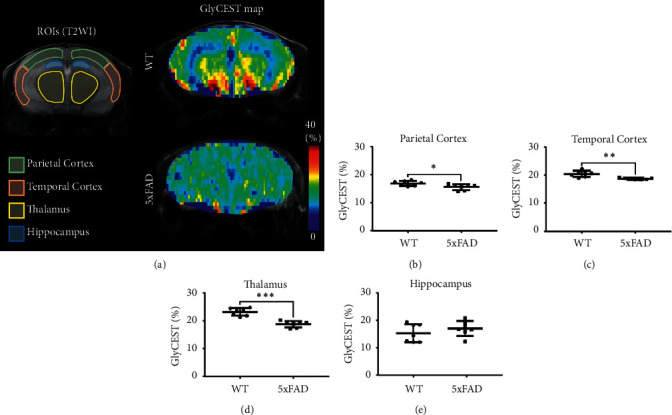
GlyCEST effects in WT (*n* = 7) and 5xFAD (*n* = 7) mice. (a) T2WI ROIs in a coronal brain slice (left panel) and the corresponding GlyCEST maps (right panel). (b–e) GlyCEST (%) of the parietal and temporal cortex, thalamus, and hippocampus. Data were analyzed using the unpaired *t*-test. ^*∗*^*P* < 0.05, ^∗∗^*P* < 0.005, and ^∗∗∗^*P* < 0.0001.

Next, we imaged GlyCEST in WT (*n* = 7) and 5xFAD (*n* = 7) mice.  Figure 4(a) shows representative GlyCEST maps in WT (upper right panel) and 5xFAD (lower right panel) mice. The GlyCEST effects in 5xFAD mice were lower in both the cortex and the thalamus compared to those in WT mice. In subregion analyses, GlyCEST (%) was significantly lower in the parietal cortex (*P*=0.0282, Figure 4(b)), temporal cortex (*P*=0.0020, Figure 4(c)), and thalamus (*P* < 0.0001, Figure 4(d)) of 5xFAD mice compared to WT animals. No differences were observed in the hippocampus (Figure 4(e)).

**Figure 5:**
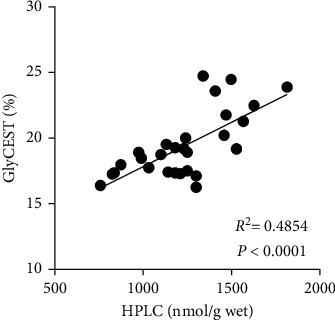
Correlation between HPLC and GlyCEST measurements. GlyCEST (%) is significantly correlated with the glycine concentration measured by HPLC.

Figure 5 shows the relationship between HPLC-determined glycine concentrations and GlyCEST (%) values in the brains of WT and 5xFAD mice. GlyCEST (%) was significantly correlated with the glycine concentration measured by HPLC (*R*^2^ = 0.4854 and *P* < 0.0001).

## 4. Discussion

Amino acid residues, such as –NH, –NH_2_, and –OH groups, exchange protons with the surrounding water molecules. By applying selective saturation pulses, it is possible to detect specific molecules within tissues, such as sugars, amino acids, transmitters, and nucleosides. While various imaging and application examples of endogenous substances, including APT CEST [22–24] and GluCEST [25, 26], have been reported, CEST imaging of glycine has not yet been described. Because glycine has a high chemical exchange rate with free water [27], a high saturation power in a short pulse time is required to image GlyCEST. High-power saturation pulses increase the specific absorption ratio (SAR) and may impose a high load on the MRI system, resulting in system limitations. In this study, a train of 10 Gaussian pulses, instead of a continuous wave pulse, was applied for presaturation to reduce the load on the radiofrequency (RF) amplifier system [28]. Through this strategy, high-power irradiation could be performed as long as the RF system allowed for it.

The GlyCEST effect of the thalamus was higher than that of the cortex in C57BL/6 WT mice. This result was confirmed by HPLC measurements. In addition, the GlyCEST maps obtained in this study were in good agreement with the findings of a previous histopathology study that examined bacterial artificial chromosome transgenic mice specifically expressing enhanced green fluorescent protein under the control of the glycine transporter 2 promoter [29].

The pathogenesis of AD has not yet been elucidated; however, the amyloid hypothesis is widely accepted [30]. This hypothesis suggests that the pathological process of AD starts with the abnormal accumulation of amyloid beta (A*β*), followed by the accumulation of tau protein, ultimately leading to neuronal death. However, in AD patients, abnormal protein accumulation does not necessarily correlate spatially and temporally with the pathology, and synaptic deficits appear at the earliest stage [31]. The NMDA receptor is a channel that controls the influx of calcium ions, and channel opening requires the binding of glycine and glutamate. A study using postmortem brain specimens from patients with AD reported that glycine levels were decreased in the superior frontal gyri of AD patients compared to those of healthy subjects [10]. In the present study, the GlyCEST effect was lower in the parietal cortex, temporal cortex, and thalamus of 5xFAD mice, supporting the findings of the previous study. A*β* aggregation increases NMDA receptor activity and induces excitotoxicity [32]. Therefore, it may suppress long-term potentiation and cause cognitive impairment [33, 34]. The cause of cortical and thalamic glycine depletion in 5xFAD mice is still unclear. However, excitotoxicity due to activation of extrasynaptic NMDA receptors may be involved [35]. NMDA receptors exist in both intrasynaptic and extrasynaptic regions of neurons. Intrasynaptic NMDA receptors regulate neural functions. However, increased activity of extrasynaptic NMDA receptors is thought to induce neuronal death due to excitotoxicity. Memantine, which ameliorates cognitive symptoms in patients with AD, is thought to exert its effects by suppressing the activity of extrasynaptic NMDA receptors [36]. Similar to glycine, D-serine is a small molecule that binds to the glycine binding sites of NMDA receptors, thereby putting NMDA receptors in a state of readiness for activation. A*β* peptides promote synthesis and release of D-serine in astrocytes through serine racemase activation. Moreover, increases in reactive astrocytes due to inflammatory responses may further upregulate D-serine synthesis [37]. Excessive D-serine release may lead to excitotoxicity by activating extrasynaptic NMDA receptors, resulting in synaptic loss and even a decrease in glycine concentration due to neural loss [38, 39]. On the other hand, the biosynthesis of glycine is maintained in equilibrium with L-serine by serine hydroxymethyl-transferase [40]. Increased activity of serine racemase by A*β* may lead to synthesis of D-serine from L-serine, resulting in decreases in both L-serine and glycine levels. On the other hand, the GlyCEST effect in the hippocampus did not differ between WT and 5xFAD mice, which is in concordance with the findings of a study in humans [41]. However, it remains unclear why the hippocampus retains glycine levels in 5xFAD mice. Our group recently reported that the dendrite/axon density of 5xFAD mice is decreased in the cortex but not in the hippocampus [26]. Similarly, a histopathological study found that in 5xFAD mice, the neuronal density of layer 5 was decreased in the cortex but retained in the hippocampus [42]. These regional differences may influence the distribution of glycine concentrations.

The present study has several limitations. The GlyCEST effect determined in this study may have some confounding factors when assessing the cerebral glycine concentration. The GlyCEST effect is pH-dependent and is enhanced under acidic conditions [27]. Other brain metabolites, magnetization transfer [43] and nuclear Overhauser enhancement [44], may also modulate GlyCEST effects. However, we confirmed the linear correlation of the GlyCEST effect with the glycine concentration using a phantom, and biochemical tests were also performed to confirm the validity of the GlyCEST method.

## 5. Conclusion

In this study, the CEST-MRI technology was proposed to map the glycine concentration distribution in the murine brain. The results demonstrated that GlyCEST imaging can quantitatively measure glycine concentrations in the brain. The GlyCEST data obtained in C57BL/6 mice showed in good agreement with the biochemical measurements that the glycine concentration in the thalamus was higher than that in the cortex. In 5xFAD mice, an animal model of AD, GlyCEST effects in the cerebral cortex and thalamus, but not in the hippocampus, were lower than those in WT mice. The present method provides a novel *in vivo* imaging tool for researchers to capture the changes in glycine concentrations.

## Data Availability

Data and program scripts used to support the findings of this study are available from the corresponding author upon reasonable request.

## Supplementary Materials

Supplementary Materials The supplementary materials present reproducibility assessments of GlyCEST measurements. Supplementary Figure S-1: GlyCEST maps and GlyCEST (%) in the cortex and thalamus from two measurements. (a) GlyCEST maps of five C57BL/6 mice (A–E). (b) The open and closed circles indicate the GlyCEST (%) average of two measurements in the cortex and thalamus, respectively. Supplementary Table S-1: GlyCEST (%) in the cortex and thalamus from two measurements and their intraclass correlation coefficients (ICCs).
