# Supplementary material for: GlyCEST: Magnetic Resonance Imaging of Glycine—Distribution in the Normal Murine Brain and Alterations in 5xFAD Mice
Source: Contrast Media Mol Imaging. 2021 Dec 30;2021:8988762. doi: 10.1155/2021/8988762 (PMC8739925; doi:10.1155/2021/8988762)
Supplement: Supplementary Materials — The supplementary materials present reproducibility assessments of GlyCEST measurements. Supplementary Figure S-1: GlyCEST maps and GlyCEST (%) in the cortex and thalamus from two measurements. (a) GlyCEST maps of five C57BL/6 mice (A–E). (b) The open and closed circles indicate the GlyCEST (%) average of two measurements in the cortex and thalamus, respectively. Supplementary Table S-1: GlyCEST (%) in the cortex and thalamus from two measurements and their intraclass correlation coefficients (ICCs). [file 8988762.f1.pdf]

Supplementary Materials

GlyCEST: Magnetic Resonance Imaging of Glycine—Distribution in the Normal Murine Brain and Alterations in 5xFAD Mice

Ken Ohno,[1,2] Masaki Ohkubo,[3] Bingwen Zheng,[4] Masaki Watanabe,[1] Tsuyoshi Matsuda,[5] Ingrid L. Kwee,[6] and Hironaka Igarashi[1]

[1] Center for Integrated Human Brain Science, Brain Research Institute, University of Niigata, Niigata 951-8585, Japan.

[2] Department of Radiological Technology, Faculty of Medical Technology, Niigata University of Health and Welfare, Niigata 950-3198, Japan.

[3] Department of Radiological Technology, School of Health Sciences, Faculty of Medicine, University of Niigata, Niigata 951-8518, Japan.

[4] Time Medical Ltd., Hong Kong Science & Technology Park, Hong Kong, China.

[5] Division of Ultrahigh Field MRI, Institute for Biomedical Sciences, Iwate Medical University, Iwate 028-3694, Japan.

[6] Department of Neurology, University of California Davis, CA 94553, USA.

Correspondence should be addressed to Hironaka Igarashi; higara@bri.niigata-u.ac.jp

a

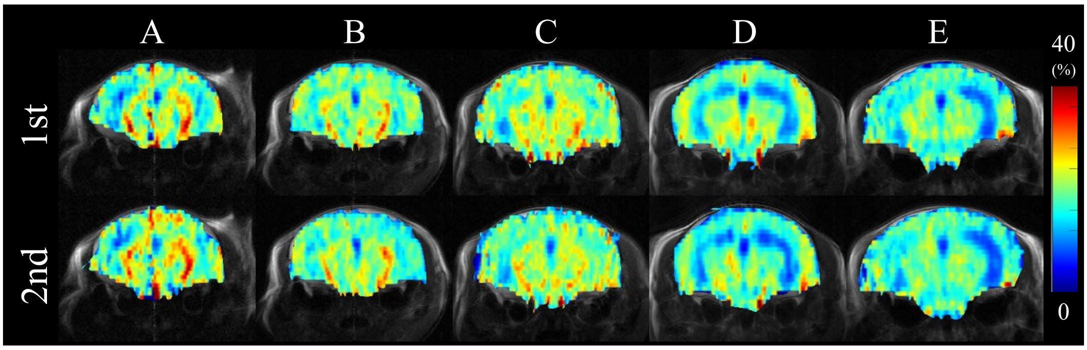

b

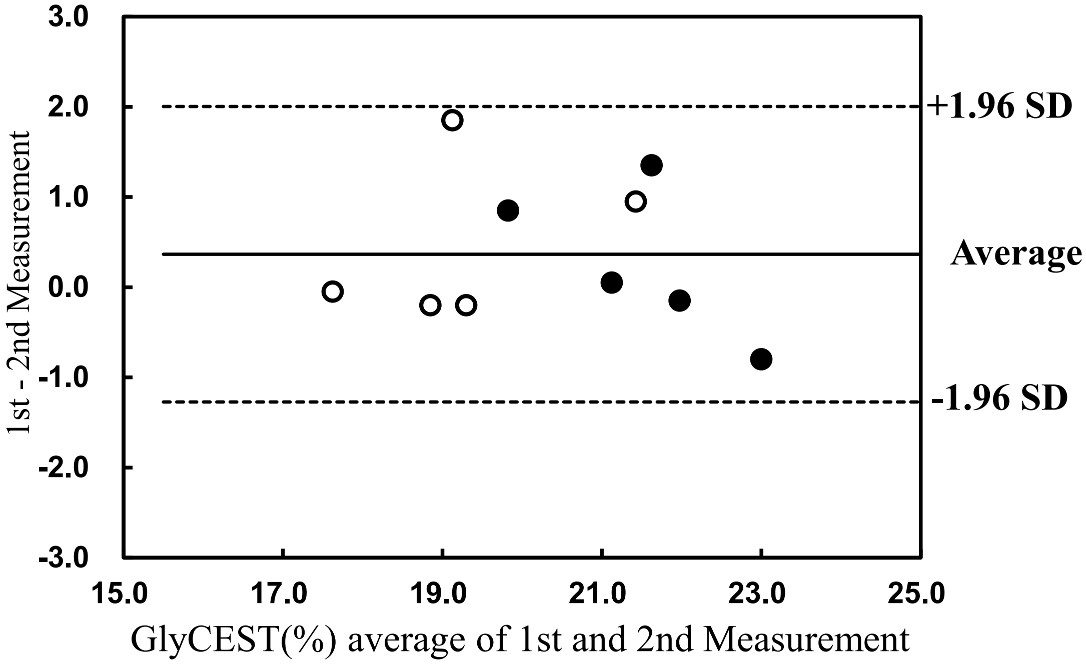

Supplementary Figure S-1: GlyCEST maps and GlyCEST (%) in the cortex and thalamus from two measurements. (a) GlyCEST maps of five C57BL/6 mice (A–E) (b) The open and closed circles indicate GlyCEST (%) average of two measurements in the cortex and thalamus, respectively.

Supplementary Table S-1: GlyCEST (%) in the cortex and thalamus from two measurements and their intraclass correlation coefficients (ICCs).

**Cortex**	**GlyCEST (%) measurement**		**ICC**	**Thalamus**	**GlyCEST (%) measurement**		**ICC**
	**1st**	**2nd**			**1st**	**2nd**	
A	18.8	19.0		A	22.6	23.4	
B	20.1	18.2		B	21.9	22.1	
C	19.2	19.4	**0.88**	C	21.2	21.1	**0.88**
D	21.9	21.0		D	22.3	21.0	
E	17.6	17.7		E	20.3	19.4	
